# Successful combination of selpercatinib and radioiodine after pretherapeutic dose estimation in RET-altered thyroid carcinoma

**DOI:** 10.1007/s00259-022-06061-8

**Published:** 2022-12-16

**Authors:** Rudolf A. Werner, Cyrus Sayehli, Heribert Hänscheid, Takahiro Higuchi, Sebastian E. Serfling, Martin Fassnacht, Maria-Elisabeth Goebeler, Andreas K. Buck, Matthias Kroiss

**Affiliations:** 1 Department of Nuclear Medicine, University Hospital Würzburg, Würzburg, Germany; 2 The Russell H Morgan Department of Radiology and Radiological Sciences, Johns Hopkins School of Medicine, Baltimore, MD USA; 3 Interdisciplinary Trial Center With ECTU and Department of Internal Medicine II, University Hospital Würzburg, Würzburg, Germany; 4 Division of Endocrinology and Diabetes, Department of Internal Medicine I, University Hospital, University of Würzburg, Würzburg, Germany; 5 Department of Internal Medicine IV, University Hospital Munich, Ludwig-Maximilians-Universität Munich, Ziemssenstrasse 5, 80366 Munich, Germany

We report on a patient affected with rearranged during transfection (RET) fusion positive papillary thyroid carcinoma (TC). After thyroidectomy and radioiodine treatment (RIT), follow-up 123I scintigraphy did not show uptake in lung nodules identified on CT (A, arrows), indicating radioactive iodine (RAI) refractory TC. Upon disease progression, the patient received the selective RET inhibitor (RETi) selpercatinib as part of an expanded access program. Diagnostic whole-body 131I scan was conducted after 15.5 months of RETi, showing intense radiotracer accumulation in sites of disease (retention after 45 h, 0.24% of the administered activity per gram of tissue mass). After RAI with 9.4 GBq, previously negative lung nodules showed intense radiotracer accumulation on post-therapeutic scan (B). Thirteen days after therapy, a peak of Tg of 2.224 ng/ml was observed, followed by a rapid decline, suggestive of tumor response (C). Eight months after first RIT, Tg dropped from baseline 148 ng/ml under TSH suppression to 21 ng/ml with CT demonstrating reduction of lung nodules (D, arrows). Another RIT using 7.5 GBq of RAI was conducted 5 months later.

Using a fixed activity of 3.7 GBq RAI, a previous case reported on treatment failure after 6 months [1]. We herein report on selpercatinib-triggered redifferentiation combined with pre-therapeutic dose estimation to increase therapeutic efficacy of RAI. This individualized approach allowed us to administer substantially higher activities (achieving tumor doses of 197 Gy). Thus, dosimetry-adjusted RAI doses may further increase anti-tumor effects, e.g., in pediatrics [2, 3].


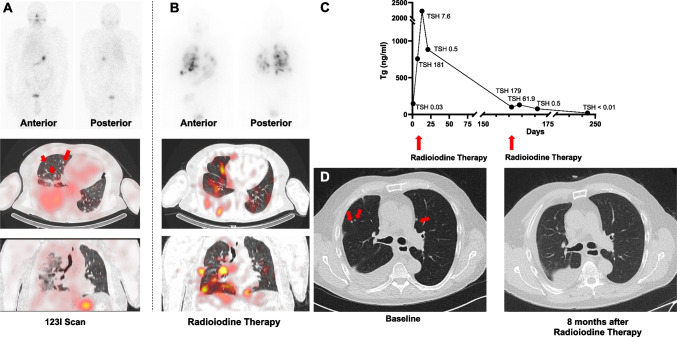


## Data Availability

Analyzed datasets are available from the corresponding author on reasonable request.
